# Inferring the impacts of evolutionary history and ecological constraints on spore size and shape in the ferns

**DOI:** 10.1002/aps3.11339

**Published:** 2020-04-20

**Authors:** David S. Barrington, Nikisha R. Patel, Morgan W. Southgate

**Affiliations:** ^1^ Pringle Herbarium University of Vermont 111 Jeffords Hall Burlington Vermont 05405‐1737 USA; ^2^ Ecology and Evolutionary Biology University of Connecticut 75 North Eagleville Road Storrs Connecticut 06269 USA

**Keywords:** ferns, genome size, ploidy level, spore shape, spore size, spore volume, spores

## Abstract

**Premise:**

In the ferns, cell size has been explored with spores, which are largely uniform within species, produced in abundance, and durable. However, spore size and shape have been variously defined, and the relationship of these traits to genome size has not been well established. Here, we explore the variation in fern spore size and shape by ploidy level and genome size.

**Methods:**

Measurements of spore dimensions for two study sets of ferns, *Polystichum* and *Adiantum*, both including diploid and tetraploid taxa, provided the basis for computing estimates of shape and size as defined here. These traits were compared between and within ploidy levels and regressed with genome size estimates from flow cytometry analysis.

**Results:**

All size traits were strongly correlated with genome size; the shape trait was weakly correlated. Tetraploids were larger than diploids as expected; the spores of some closely related diploid species were also significantly different in size.

**Discussion:**

Researchers with access to a student‐grade compound microscope can develop a valid estimate of relative genome size for ferns. These estimates provide enough resolution to infer ploidy level and explore the relationship between genome size, habitat, and physiological constraints for species within ploidy level.

The importance of genome size as a witness to the ecological and historical features of plant species has become increasingly recognized (Cui et al., 2006; Soltis et al., 2015). Critical to determining genome size is the technique of flow cytometry; this method has become easier to implement in recent years, but logistical and financial constraints still present barriers to the development of insights into genome size in many situations (Leitch and Bennett, 2007; Bainard et al., 2011). Proxies for genome size requiring little laboratory infrastructure and minimal funding are available and have a long history in plant biology—these include most prominently the size of single cells with determinate growth including stomates, pollen, and spores (Sax and Sax, 1937; Manton, 1950; Beaulieu et al., 2008).

In the ferns, spore size has long been useful for systematic and evolutionary studies; early work includes the comparative quantitative analysis of Weaver (1896). Wagner (1974) provided a substantial overview of the factors impacting spore size in the ferns, with attention to environmental factors and reproductive biology as well as ploidy. A frequently cited paper by Barrington et al. (1986) set the stage for the modern development of quantitative methods for analyzing spore size differences among closely related species, as well as the introduction—for monolete spores—of spore volume as a measure of spore size difference. Recent work has also included statistical analysis of spore size data as a standard component of the strategy used to infer the evolutionary histories of lineages, including polyploids in the ferns (Grusz et al., 2009; Beck et al., 2010; Sigel et al., 2011). Much of the potential utility in measuring spore size remains unexplored, and there are virtually no quantitative data on the shape of fern spores in the literature. Spore size, as represented in the literature until now, conflates size and shape because of the common practice of providing the measurement of only one dimension per spore. In the case of monolete spores, the “size” measure is actually length; in the case of trilete spores it is the longest dimension, sometimes referred to as the width of the spore.

Here we define spore size as the space occupied by the spore. A set of measurements in one dimension—length, width, and height—provides the most convenient basis for calculating size. Although volume seems likely to be the best trait to use in assessing the relationship between spore size and genome size, it may not be better than one of its component measurements in representing genome size. Nevertheless, volume has value in that it enables size comparison between taxa with monolete versus trilete spores. We define spore shape to be its external form, including but not limited to the two major types of form: monolete and trilete. We have found that the simplest representation of shape is the ratio of two different measurements; a single dimension is not sufficient to represent shape. Virtually nothing is known of the determinants of spore shape.

At the present time, there are virtually no data on size estimated from volume calculations for monolete spores, and none for trilete spores. Similarly, ratio data to quantify spore shape are scant. In a few cases, spore size data, typically measurements of the longest dimension, have been presented along with genome size data (Ekrt and Stech, 2008; Dyer et al., 2012; Chang et al., 2013; Ekrt and Koutecký, 2015; Patel et al., 2018; Southgate et al., 2019). These contributions suggest that spore size, whether represented by volume or its component measurements, has real potential to serve as a proxy for genome size, warranting further investigation to refine quantitative measurements of spores.

Whereas differences in spore size between species of different ploidy levels can inform an understanding of the systematic relationships among species in polyploid complexes, spore size disparity between species of the same ploidy level may be related to other factors including attributes of their ecological niches and geographic distributions (Carlquist, 1966; Barrington et al., 1986). Variation in environmental factors may lead to the evolution of differences in spore size between closely related species or different populations of the same species. For example, Cox and Hickey (1984) observed differences in megaspore size between populations of *Isoetes storkii* T. C. Palmer along several environmental gradients in Costa Rica. These differences can potentially confound the relationship between spore size and ploidy level among closely related taxa.

Here, we explore the potential utility of using spore measurements and the size and shape traits (volume and length : width ratio) calculated from them to test systematic and ecological hypotheses with two study sets developed by our research group at the University of Vermont (Fig. 1). The *Adiantum pedatum* L. complex, a clade of maidenhair ferns with trilete spores, consists of two diploid taxa, *A. aleuticum* (Rupr.) C. A. Paris and *A. pedatum*, as well as their allotetraploid derivative *A. viridimontanum* C. A. Paris. The *Polystichum californicum* (D. C. Eaton) Diels complex, a group with monolete spores, comprises the tetraploid *P. californicum* and four diploid taxa: the twice‐pinnate diploid *P. dudleyi* Maxon (certainly one progenitor of the tetraploid) and three once‐pinnate taxa. The once‐pinnate taxa, any of which may be the other progenitor of the tetraploid, are: *P. munitum* (Kaulf.) C. Presl and two subspecies of *P. imbricans* (D. C. Eaton) D. H. Wagner, subsp. *imbricans* and subsp. *curtum* (Ewan) D. H. Wagner. For both of these clades, we present two approaches to quantification of spore traits. (1) We explore the utility of spore size with both spore volume and its component measurements. In the process, we inaugurate a measure of spore volume for our tests in the case of trilete spores. (2) We report shape as estimated from ratios of measurements of single dimensions, and comment on the variation in spore shape among closely related species. The result is the basis for making inferences about genome size as part of ecological and evolutionary studies in the ferns without access to a flow cytometer.

**Figure 1:**
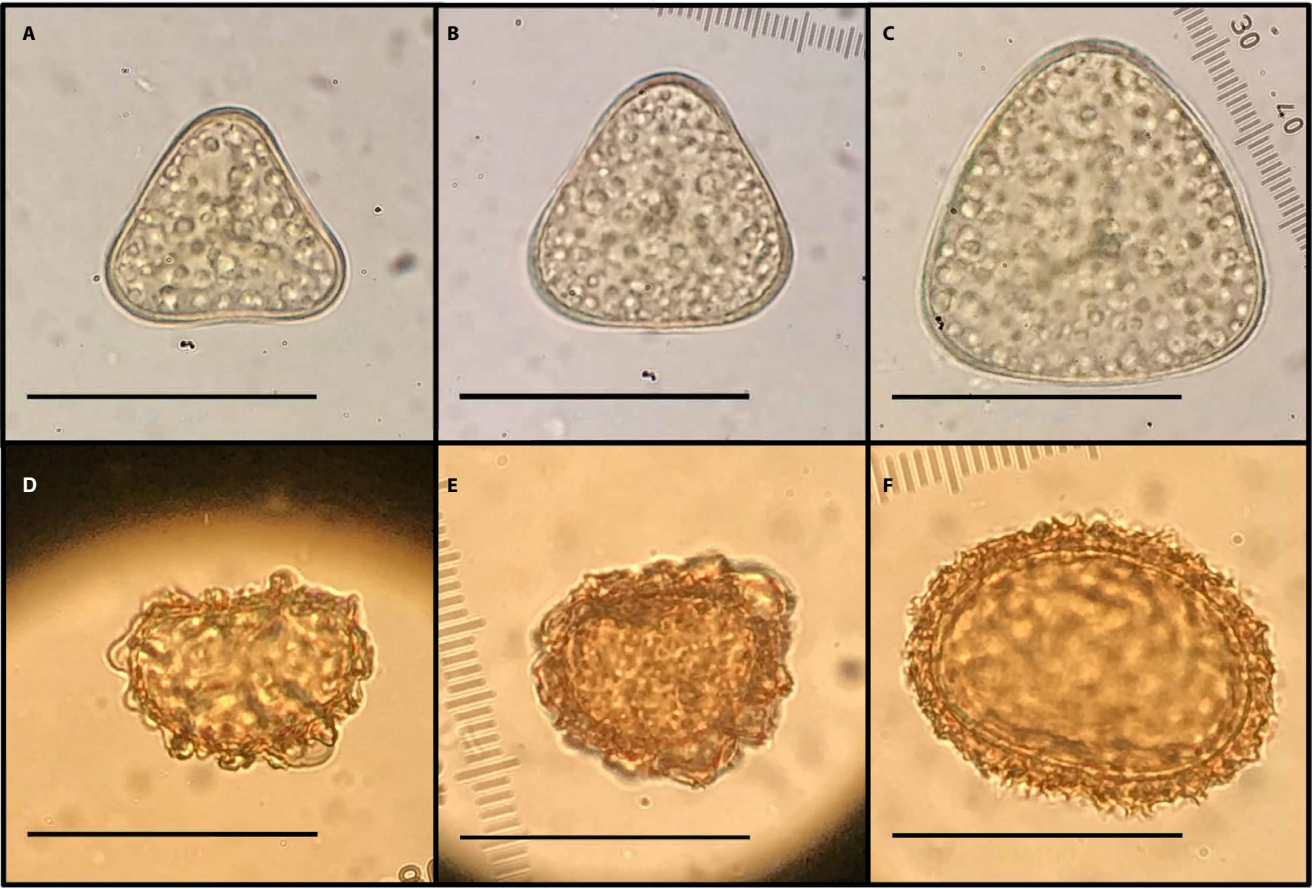
Example spores from the two study sets. Trilete *Adiantum* spores: *A. pedatum* (A), *A. aleuticum* (B), and *A. viridimontanum* (C). Monolete *Polystichum* spores: *P. imbricans* subsp. *curtum* (D), *P. dudleyi* (E), and *P. californicum* (F). All images are scaled to the same size, with scale bars = 50 μm.

We are particularly interested in the relationship between spore size and dispersibility of species restricted to rare and discontinuous habitats. Our case study of *Adiantum* L. provides the opportunity to compare spore size between two closely related diploid species with remarkably different distributions and ecological niches. Whereas *A. pedatum* is widely distributed in rich woods, a common habitat in northeastern North America, *A. aleuticum* is restricted to serpentine outcrops and serpentine forests, exceedingly rare habitats in the region (Paris, 1991; Southgate et al., 2019). Citing the hypotheses for forest patches on oceanic islands (Carlquist, 1966), Barrington et al. (1986) hypothesized that the spores of *A. aleuticum* are significantly larger than those of *A. pedatum* because successful spore germination and gametophyte establishment for this species is likely contingent upon local dispersal within a given island of serpentine habitat. They found spore size of the two diploids to differ significantly in a way that led them to accept the hypothesis. We revisit this same question here.

We consider similar hypotheses in our case study of the three once‐pinnate western North American *Polystichum* Roth taxa, because they differ in habitat parameters including substrate, insolation, and companion trees (Wagner, 1979).

## METHODS

### Sampling

The composite sample comprised spores of taxa in the two study sets (the *A. pedatum* complex and western North American *Polystichum*—all spores were collected from the specimens cited in Appendix 1; details of sample size and statistical data are provided in Appendices S1 and S2). For each group, the spore size data are part of an evolutionary study for which flow cytometry data, as well as additional data sets, were combined in previous work from our research group (Schmeckpeper, 2016; Southgate et al., 2019). In the present study, spore measurements generated during each previous study were augmented with the addition of spores from other accessions, and the combined measurements were analyzed to provide novel estimates of spore size and shape. Based on previous cytological work (Wagner, 1973; Paris and Windham, 1988), all of the species included in this study are known to have sexual (as opposed to apomictic) reproduction.

### Preparation of spores

Spores of *Adiantum* species were obtained for measurement by tapping the abaxial side of sporulating tissue onto a microscope slide under a dissecting scope and gently crushing the sori to release spores if necessary. For the western North American *Polystichum* sample, spores were harvested by inserting a probe coated with Hoyer's medium into a sorus with dehisced sporangia, then dispersing the harvested material into Hoyer's medium on a microscope slide. All spores were visualized using an Olympus BH‐2 compound microscope (Olympus Corporation, Center Valley, Pennsylvania, USA) at 400× or 600× magnification and photographed with a smartphone camera (LG V30, LG Electronics, Seoul, South Korea) and mount system (Gosky Universal Cell Phone Adapter Mount, Gosky Optics, Atlanta, Georgia, USA). Spores with irregular shape were excluded from measurement. For *Adiantum*, irregular spores were diagnosed by (1) absence or irregularity of their sporopollenin coat, making them transparent to some degree, and (2) by a drastic departure from the typical trilete shape. For *Polystichum*, spores were excluded as irregular if they were not ellipsoidal.

Spore measurements were performed in ImageJ (Schneider et al., 2012). Calibration was performed using an ocular micrometer calibrated with an object micrometer.

### Orientation and choice of spores for imaging

**Figure 2:**
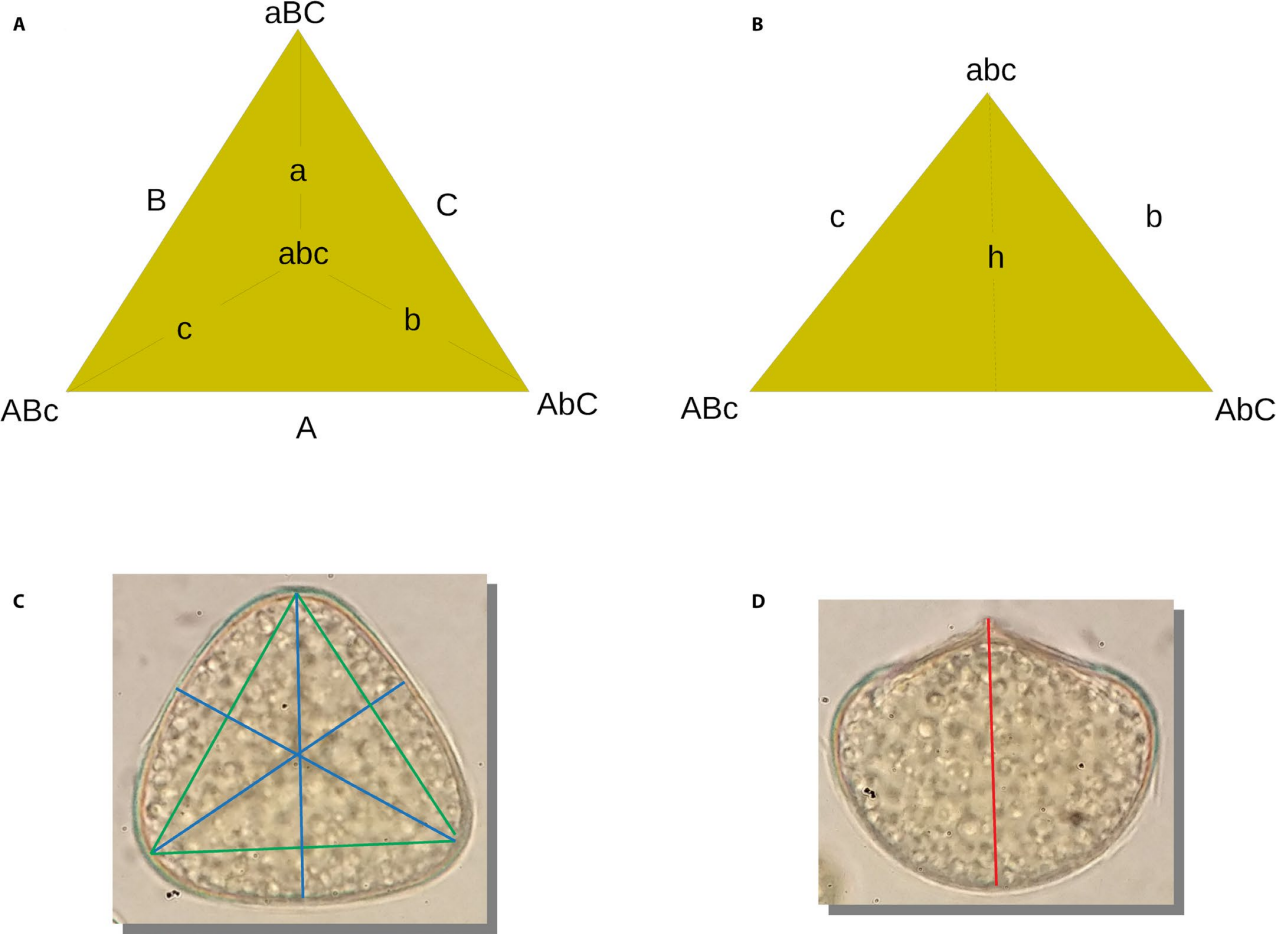
Diagram and images of the proximal (A, C) and equatorial (B, D) aspects of a trilete spore. (A) Diagram of the proximal aspect of a trilete spore; the proximal edges are labeled with lowercase letters, and the distal edges with uppercase. Vertices are indicated with the letters of the three edges that converge there. (B) Diagram rotated 90° in view from A, showing a trilete spore in equatorial aspect; the height from the proximal vertex to the center of the distal face is labeled *h*. (C) Proximal aspect of a trilete spore of *Adiantum* with the trilete mark centered, showing the three width measurements (blue) and edge measurements (green). (D) Equatorial aspect of a trilete spore of *Adiantum*, showing the height measurement (red).

For trilete spores, two orientations are required to calculate spore size and shape: (1) a proximal view with the trilete mark centered in the visible face of the spore or with all edges of the spore uniformly in focus, and (2) an equatorial view rotated 90° from the first view to reveal the height from the center of the trilete mark (the proximal vertex) to the distal face of the spore (Fig. 2). To obtain these two views, individual spores were rotated to the correct aspect by gently pushing on the edge of the cover slip with a probe. The choice of imaged spores for measurement is critical to reduce variance. Therefore, only the spores meeting these two conditions were measured and analyzed.

For monolete spores, a single orientation is sufficient, but again, choice of spores is critical. Because not all spores have their long axis parallel to the plane of the slide, spores appearing round were excluded, presuming they were oriented with the long axis perpendicular to the slide. Among the remaining spores, those with the laesura visible and in the same focal plane throughout and those with the laesura not visible but the exospore at the two ends in the same focal plane were included in the sample.

### Spore measurement: trilete spores

We inaugurated a system for naming the vertices and edges of trilete spores to enable repeatable measurements yielding values for volume as well as shape (Fig. 2). The six edges of the spore are designated by letters, with proximal edges identified by lowercase (*a,b,c*) and distal edges with uppercase (*A,B,C*). Edges labeled with the same letter (e.g., *a* and *A*) comprise a proximal edge and the distal edge that is perpendicular to it, and vertices are designated by the letters of their three edges (e.g., *aBC*). The vertex with the trilete mark is the only one named solely with lowercase letters (*abc*) because the proximal ends of the three proximal edges make up this vertex. Using this naming system, six measurements of spore dimensions were taken in the proximal view and one measurement in the equatorial view. The perispore was included in all measurements, as it is thin and unornamented in the *Adiantum* species. First, in the proximal view, three width measurements were taken across the trilete face (e.g., *ABc‐C*) and averaged to produce a single width measurement. Second, also in the proximal view, the lengths of the three proximal edges were measured (*A*,* B*, and *C*). Third, in the equatorial view, spore height was measured as the length from the center of the trilete mark to the center of the distal face. Using the measurement of spore height in conjunction with the edge measurements and individual width measurements, the lengths of the three edges proximal to the trilete mark (e.g., *a*,* b*, and *c*) were calculated as the hypotenuse of a right triangle using the Pythagorean theorem. To do so, each individual width measurement (e.g., *ABc‐C*) was multiplied by two‐thirds in order to estimate the length from the distal vertex (e.g., *ABc*) to the point on the distal face directly below *abc* (e.g., the right angle of the triangle). So, for example, the length of edge *c* was calculated as *c* ^2^ *= h* ^2^ *+* (⅔*ABc‐C)^2^.

Incorporating the length of the three measured and the three calculated edges of the trilete spore, the value for spore volume was calculated as the volume of an irregular tetrahedron (formula obtained from https://keisan.casio.com/exec/system/1329962711), because the six edges of trilete spores are not always equivalent in length. Using this formula to estimate trilete spore volume is an imprecise estimate because the bottom of trilete spores is rounded rather than flat.

For the trilete spores of *Adiantum*, we calculated the ratio of width to height to represent shape.

### Spore measurement: monolete spores

A single length measurement (the longest dimension of the spore) and width measurement (perpendicular to and at the midpoint of the length measurement) were taken for the exospore of each monolete spore (we excluded the elaborate perispore). The value for spore volume was calculated using the formula V = πLW^2^/6 (following Barrington et al., 1986), for which monolete spores are considered ellipsoids. As with trilete spores, the formula for an ellipsoid to estimate monolete spore volume is an imprecise estimate, because the monolete spores are not bilaterally symmetrical along the long axis and they are curved at maturity.

For the monolete spores of *Polystichum*, we calculated the ratio of length to width to represent shape.

### Genome size estimation

Genome size estimates for each species were obtained from data collected by our lab group. For the *Adiantum* species, data published in Southgate et al. (2019) were supplemented with additional data. For the *Polystichum* species, these data come from Schmeckpeper (2016). For the majority of specimens included in the *Polystichum* sample, flow cytometry was performed on the same specimens. For all specimens included in the *Adiantum* sampling, associated genome size estimates are derived from averages for the species.

Tissue preparation for flow cytometry for yielding all data here follows the protocol of Bainard et al. (2011), but with either propidium iodide or Sybr Green (Life Technologies, Carlsbad, California, USA) as the fluorophore. The leaf tissue was placed in a Petri dish, and 1.2 mL of LBO1 buffer (Galbraith et al., 1997), 100 μL of RNase, and 10 μL of DNA‐specific dye were added. The tissue was finely chopped in solution and strained through a 30‐μL filter. The flow‐through of released nuclei was analyzed within 2 h of preparation. Fresh tissue of *Pisum sativum* cv. Ctirad, with a known 2C value of 9.09 pg (Doležel et al., 1998), was utilized as an external or internal genome size standard for *Adiantum*. For *Polystichum*, fresh tissue of *Hordeum vulgare* L., with a known 2C value of 11.1 pg (Bennett and Smith, 1976), was utilized. For use as an external standard, tissue was prepared separately from the sample tissue following the same protocol. For use as an internal standard, equivalent masses of standard and sample tissue were combined and processed following the same protocol. Samples were analyzed using a Coulter Epics XL flow cytometer (Beckman Coulter Genomics/Genewicz, South Plainfield, New Jersey, USA) maintained at the Plant Biology Department, University of Vermont. Histograms of the event numbers were analyzed using FlowJo (Treestar Inc., San Carlos, California, USA). The genome size of each sample was estimated using the following formula: sample 2C value (DNA pg) = (reference 2C value × sample 2C mean peak position) / reference 2C mean peak position. We report genome size values in picograms (pg).

Genome size inferences based on flow cytometry data associated with *Adiantum* samples are based on samples represented by ~2500 events, on average, and the average coefficient of variance for the event peaks was 7.6. For the *Polystichum* data set, flow cytometry data associated with samples were analyzed for genome size inference if fluorescent peaks comprised at least 200 events with a coefficient of variance less than 15.

### Spore data analysis

Summary statistics (including the mean, standard deviation, and coefficient of variance) for the spore measurements and the values calculated from them were calculated by accession (Appendix S2) and by species (Tables 1, 2). The data were further analyzed on two levels, consistent with the two possible scenarios in which spore measurements can be utilized—with species identifications only, or with an estimate of genome size. First, ANOVA was used to assess the difference in spore size and shape traits among all the species within each study set, followed by a Tukey post‐hoc test to assess the differences between all possible pairs of species. Second, to assess the relationship between spore traits and genome size, we conducted a rank correlation test, because our spore data, although approximately normal in distribution by species for each trait, were not normally distributed by trait within each complex. Spores are presumed haploid in the sexual fern life cycle, and so to associate genome size with spore size and shape, the haploid (1C) genome size estimates were used as input values in all statistical analyses. Finally, the relationship between spore volume (the only trait directly comparable between trilete and monolete spores) and genome size was assessed for the data combined by complex. The composite spore‐volume data showed a log‐normal distribution, and therefore were base‐*e* log‐transformed prior to analysis. A linear regression of the log of spore volume as a function of genome size showed heterogeneity of variance between the *Polystichum* and *Adiantum* study groups, and so clade was added to the linear model as an interaction term.

**Table 1:** Mean spore size and shape trait values by species in the western North American *Polystichum* clade.^a^

Taxon (Ploidy)	Diploid genome size, pg	Length, μm	Width, μm	Length : width	Volume, μm^3^
*Polystichum munitum* (2*n*)	11.85	32.9 (0.6, 11.5)	25.3 (0.6, 14.9)	1.31 (0.10, 7.8)	11,592 (743.8, 40.6)
*Polystichum dudleyi* (2*n*)	No data	34.9 (0.5, 7.7)	25.9 (0.3, 6.6)	1.35 (0.10, 7.2)	12,380 (430.8, 18.1)
*Polystichum imbricans* subsp. *imbricans* (2*n*)	16.17	37.26 (0.6, 11.2)	27.7 (0.6, 13.7)	1.36 (0.14, 10.3)	15,538 (798.1, 33.2)
*Polystichum imbricans* subsp. *curtum* (2*n*)	13.16	40.4 (0.5, 11.3)	30.3 (0.5, 15.1)	1.34 (0.12, 8.9)	20,399 (813.4, 35.9)
*Polystichum californicum* (4*n*)	21.27	49.0 (0.72, 9.35)	37.6 (0.61,10.28)	1.31 (0.08, 5.95)	37,200 (1672, 28.4)

Standard error and coefficient of variance (in %) are shown in parentheses after each mean.

**Table 2:** Mean spore size and shape trait values by species in the *Adiantum pedatum* complex.^a^

Taxon (Ploidy)	Diploid genome size, pg	Average width, μm	Height, μm	Width : height	Volume, μm^3^
*Adiantum pedatum* (2*n*)	8.47	35.1 (0.3, 5.3)	30.9 (0.3, 5.1)	1.14 (0.03, 3.02)	6026 (145, 13.8)
*Adiantum aleuticum* (2*n*)	9.92	39.6 (0.5, 6.8)	34.4 (0.4, 6.6)	1.15 (0.03, 2.81)	8349 (300, 19.7)
*Adiantum viridimontanum* (4*n*)	21.1	51.2 (1.0, 10.8)	43.8 (1.0, 12.4)	1.17 (0.04, 3.72)	17,730 (1137, 34.5)

a Standard error and coefficient of variance (in %) are shown in parentheses after each mean.

## RESULTS

### Western North American *Polystichum*

Spores of diploid western North American *Polystichum* species ranged in length from 32.9 μm in *P. munitum* to 40.4 μm in *P. imbricans* subsp. *imbricans*, whereas the tetraploid *P. californicum* had a mean spore length of 49.0 μm (Table 1). Similarly, spores of the diploid species ranged in width from 25.3 to 30.3 μm, whereas tetraploid *P. californicum* had a mean width of 37.6 μm. Overall, shape as represented by length : width ratio was quite uniform. The diploids ranged widely in volume: the largest was *P. imbricans* subsp. *curtum* with a mean spore volume of 20,399 μm^3^; the smallest was *P. munitum*, with a mean of 11,592 μm^3^. The tetraploid *P. californicum* had a mean volume of 37,200 μm^3^ (Table 1).

Differences in spore size among species in the *Polystichum* study set were highly significant whether estimated from volume or its component measurements, whereas the difference in shape as represented by the length : width ratio was insignificant (Appendix 2). Differences between polyploid *P. californicum* and each of the four diploid taxa were all highly significant for volume as well as the component size measurements, but insignificant for the shape trait (the length : width ratio) (Appendix 3). Within the diploids, although the differences between the once‐pinnate taxa were in general highly significant for volume and its component measurements, two of them (excluding *P. imbricans* subsp. *curtum*, the largest) were not significantly different from the twice‐pinnate diploid *P. dudleyi* in any of the spore traits*. Polystichum munitum* and *P. imbricans* subsp. *imbricans* were significantly different in length and width but not significantly different in volume (Appendix 3).

The coefficient of variance for the length : width ratio was low (6% for the polyploid, 7.2–10.3% across all diploids), documenting uniform shape across the sample (Table 1). By contrast, the coefficient of variance for volume was much higher (18.1–40.6% across the diploids, 28.4% for the polyploid), revealing substantial variation in the size of the spores as estimated by volume.

Variation in size was similar by species and by accession for *Polystichum* (Table 1, Appendix S2). In contrast, variation in shape was much lower by accession than by species.

### Northeast North American *Adiantum*

For volume and its component measurements, the spores of *A. pedatum* were smallest, the spores of *A. aleuticum* intermediate, and the spores of *A. viridimontanum* largest (Table 2). In terms of average width, the spores of *A. pedatum*,* A. aleuticum*, and *A. viridimontanum* averaged 35.1, 39.6, and 51.2 μm, respectively. The average spore height was calculated to be 30.9, 34.4, and 43.8 μm for *A. pedatum*,* A. aleuticum*, and *A. viridimontanum*, respectively. The average spore volume was 6026, 8349, and 17,730 μm^3^ for *A. pedatum*,* A. aleuticum*, and *A. viridimontanum*, respectively. For the width : height ratio, the spores of *A. pedatum*,* A. aleuticum*, and *A. viridimontanum* averaged 1.14, 1.15, and 1.17, respectively.

For all four spore traits assessed here, *A. viridimontanum* had the highest coefficient of variance. Of these traits, spore volume exhibited the highest coefficient of variance for the three species, with values of 13.8%, 19.7%, and 34.5% for *A. pedatum*,* A. aleuticum*, and *A. viridimontanum*, respectively (Table 2). As in *Polystichum*, volume within taxon varied more than shape in *Adiantum*. Variance by accession for all spore traits was comparable to variance by species (Table 2, Appendix S2).

We found that spore size can be distinguished by the original measurements as well as by volume; all traits were significantly different among the three *Adiantum* species (Appendix 4). Comparing species pairwise, all differences were highly significant for average spore width and spore height (Appendix 5). For the width : height ratio, the difference was significant between *A. pedatum* and both serpentine maidenhair ferns, but not significant between *A. aleuticum* and *A. viridimontanum*. The difference in spore volume was highly significant between *A. viridimontanum* and both of its progenitors, and significant between *A. aleuticum* and *A. pedatum*.

### Spore traits and genome size

For both study sets, the correlation between volume, its component measurements, and genome size was highly significant, with genome size explaining more than 85% of the variation in spore size traits (Table 3). For western North American *Polystichum*, the correlation coefficient was highest for spore volume, second highest for spore width, and third highest for length. For the *Adiantum* complex, the correlation coefficient was highest for the average width, second highest for volume, and third highest for height (Table 3). The correlation between spore shape and genome size was also significant, with the correlation coefficient explaining about one‐third of the variation in both study sets (Table 3).

**Table 3:** Coefficients of correlation (*ρ*) between genome size and spore trait values for western North American *Polystichum* and the *Adiantum pedatum* complex.^a^

*Polystichum*	Spore trait	Width	Length	Length : width	Volume
	*ρ*	0.877***	0.868***	−0.372**	0.885***

*Adiantum*	Spore trait	Average width	Height	Average width : height	Volume
	*ρ*	0.899***	0.887***	0.345**	0.891***

^a^ Three asterisks denote significance at *P* < 0.001, whereas two denote significance at *P* < 0.01.

**Figure 3:**
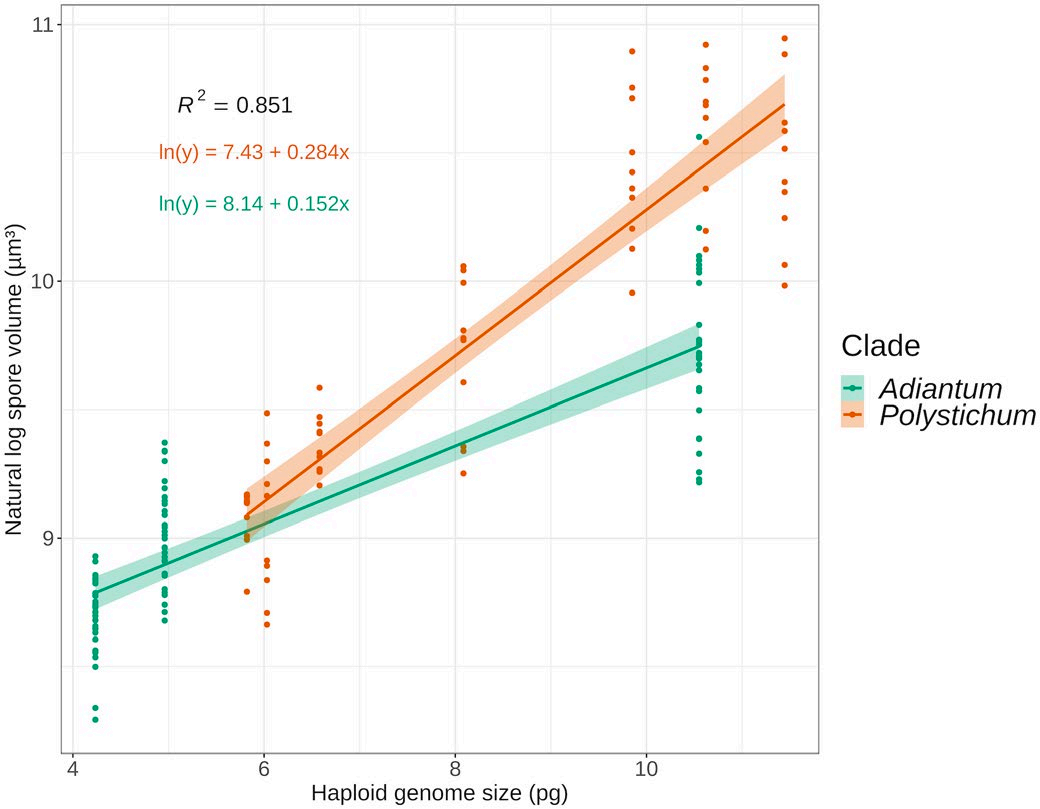
Linear regression model of the base‐*e* log of spore volume as a function of the interaction between haploid genome size and clade, for the combined *Adiantum* and *Polystichum* study sets. The fit for each clade is shown separately, with 95% confidence intervals. Model fits for each clade are shown in log‐transformed units.

In the linear regression model, the interaction between haploid genome size and clade explained 85.1% of the variation in spore volume (Fig. 3). The analysis of covariance found a significant difference between the slope coefficients of each clade, with *P* < 0.001. In normal rather than base‐*e* log‐transformed units, the coefficient for *Adiantum* was estimated at 1.16 (±0.02) and the coefficient for *Polystichum* was estimated at 1.33 (±0.04). Thus, for each picogram increase in genome size, our model predicts a percent increase in spore volume of approximately 16% for *Adiantum* and 33% for *Polystichum*.

## DISCUSSION

In this contribution, we have focused on the variation in spore size and shape across ploidy level, species, and habitat preference by exploring four specific inquiries: (1) Can spore size be used in the absence of flow cytometry data to illuminate ploidy level in the ferns, thus providing insight into reticulate evolutionary histories? (2) Can spore size be used within ploidy level to distinguish species, thus opening the way for discovering the role of spore size as it may relate to adaptation to ecological niche? (3) Are there patterns to spore shape, and are there possible biological explanations for these patterns? (4) What is the relationship between spore volume and genome size across disparate phylogenetic clades? Here we summarize the outcomes of our inquiry and consider their implications for understanding the evolution and ecology of ferns.

### Spore size and shape in the resolution of ploidy level

Historically, spore size, variously defined, has been considered alone as a proxy for ploidy level. Here, we have distinguished spore size from spore shape and tested the utility of each as a proxy for genome size in light of flow cytometry data, and found that distinguishing the two is important in ways that differ for our two study sets. In both study sets, we have demonstrated that spore size traits vary significantly between closely related species of different ploidy levels (Tables 1, 2). Size, best represented by volume, length (for monolete spores), or width (for trilete spores), distinguishes taxa in these study sets much better than shape as represented by a ratio of two linear measurements. We found no advantage to calculating volume in this context, because the one‐dimensional measurements of spore size are just as powerful at distinguishing species.

Coefficients of variance calculated for the two groups revealed patterns of potential use in understanding the determinants of spore size and shape in the ferns. In both groups, the coefficient of variance for size was much higher than that for shape (Tables 1, 2). Thus, size is more variable in these groups than shape. In fact, shape is remarkably uniform within as well as between species here, although more so for *Polystichum* than for *Adiantum*. In *Adiantum*, the coefficient of variance for size was higher in the polyploid than in the diploids, in concert with previous reporting that polyploids varied more in size (Wagner, 1973). However, the polyploid *P. californicum* did not have a coefficient of variance for spore size higher than the diploids.

### Spore size, dispersibility, and ecological niche

Spores of the diploid species *A. aleuticum* and *A. pedatum* were different in size from one another, with the former producing significantly larger spores as evidenced by average width, height, and volume (Appendix 5). Considering the ecology of the two species, *A. pedatum*, which occupies a continuous habitat, produces smaller propagules than *A. aleuticum*, which occupies a discontinuous habitat. Consequently, our results lend support to the idea first presented by Barrington et al. (1986) that in northeastern North America *A. aleuticum*, which is restricted to patchily distributed serpentine outcrops, may have evolved larger spores than its congener as an adaptation to limit dispersal to the unfavorable surrounding forests. This explanation is consistent with Carlquist's hypothesis regarding the pattern of spore size variation in continuous and discontinuous Hawaiian habitats (Carlquist, 1966).

A similar pattern is evident among the diploids in the western North American *Polystichum* study set. The diploids from the more continuous forests, *P. munitum* and *P. dudleyi*, have smaller spores; those from the drier, more open, and patchier forest habitats have larger spores. Similarly, Wagner (1973) reported higher spore size ranges, relative to other western *Polystichum* diploids and tetraploids, in what is now *P. lemmonii* Underw. (diploid), common on patchy serpentine substrates, and in its tetraploid derivative *P. scopulinum* (D. C. Eaton) Maxon, also common on serpentine soils.

For both groups examined here, variation in spore size within ploidy levels as well as within species suggests that factors other than genome size also play a role in spore size. Although spore size has clear utility in resolving ploidy level within small taxonomic groups, determinations of ploidy may be complicated by an array of factors. Reproductive biology, ecology, and the spore size of hybrid‐progenitor species must all be considered when drawing conclusions about ploidy level. In turn, the variation in spore size not explained by variation in genome size has the potential to provide evidence of evolutionary, ecological, and historical patterns of interest for the clade in question.

### Relationship between spore dimensions and genome size

Our results provide quantitative confirmation that genome size is a fundamental determinant of spore size for fern species with both monolete and trilete spores. In both the *A. pedatum* complex and western North American *Polystichum*, we found strong correlations between all three spore size trait values and genome size, with the former explaining approximately 86–90% of the variation in the latter (Table 3). Thus, researchers without access to a flow cytometer can proceed with the working hypothesis that spore size traits are a proxy for genome size. We found that the correlation of spore size measurements with genome size is as significant as the volume calculated from them for both clades, but we recommend reporting volume because of its more direct relationship with genome size and because it enables comparison of spore size between disparate phylogenetic groups.

Our linear model of spore volume as a function of the interaction between genome size and clade shows that this relationship is comparable between clades, even those with very different spore architectures (Fig. 3). However, the model found significantly different slopes between the *Adiantum* and *Polystichum* study sets; the effect of increased genome size on spore size is greater for *Polystichum* than for *Adiantum*. There are several possible explanations for this pattern: the correlation may differ on the basis of spore architecture or by some other biological attribute that differs across these groups.

We also found a slight correlation between spore shape and genome size for both study sets. The width : height ratio of *Adiantum* spores is higher in the tetraploid than in the diploid species; in *Polystichum*, the length : width ratio for the tetraploid is lower than three of the four diploid species. Evidently, we have much to learn about spore shape.

### Sources of error and improvements

There are some potential sources of methodological error to consider in the protocols presented here; first, we consider challenges to developing a data set of precise, accurate measurements. With careful choice, spore images will exclude spores at oblique angles. Nonetheless, selected spore images nearly approach but do not attain perfect polar or equatorial views. For genera with highly ornamented perispores, measuring exospore dimensions eliminates substantial variance. However, in practice, the consistent identification of the limit of the exospore is difficult enough to introduce variance. Similarly, defining a scale using a micrometer included in the image is a source of error if the focus of the ocular micrometer is different among samples. With regard to our volume formulas, trilete and monolete spores are not truly irregular tetrahedrons and ellipsoids; hence, the true volumes differ from those presented here. Fortunately, using a consistent approach (i.e., the same measurements and formulas throughout) makes comparisons within a single study group possible; comparing studies presents additional challenges if the methods differ. Finally, considering the statistical analyses, a broader taxonomic sampling that evaluates correlations between spore dimensions and genome size employing phylogenetically independent contrasts may reveal greater or less utility of the spore traits presented here.

### Suggestions for future application

Depending on access to equipment, researchers may (1) generate and use flow cytometry data in conjunction with spore size and shape data, (2) use spore size data in conjunction with published genome size estimates for their study set, or (3) measure spores alone. In each case, inferences about ploidy can only be made within a discrete group of closely related taxa that ideally constitute a clade. More studies utilizing the first approach, especially ones that report spore size and genome size for the same accession, would likely increase the feasibility and usefulness of studies implementing the second and/or third approach, widening the scope in which spore size data can be used to inform the construction of evolutionary hypotheses. The third approach to inferring ploidy should ideally be undertaken in conjunction with other data sets—such as morphometric data, data from phylogenetic analysis, and spore counts—in order to make parsimonious inferences about ploidy that account for reproductive mode and take morphological intermediacy into account (for allopolyploid taxa). Without access to genome size data for the species of interest, hypotheses about ploidy levels within a clade can still be formed, but actual genome size cannot be estimated because there does not at present appear to be a way to estimate DNA content in picograms from measures of spore volume.

We have reported little evidence of a significant difference in spore shape across ploidy levels in this study; there is no relationship at all in the *Polystichum* taxa and marginal significance at best in the *Adiantum* study set. However, at least one study suggests that some fern lineages have significant relationships between shape and ploidy. Braithwaite (1964), in the process of proposing a second apomictic life cycle in the ferns, charted length : width ratios (shape) against length (size) from 50 spores for each of four *Asplenium* species: three sexual species (a tetraploid, an octoploid, and a dodecaploid) and one apomictic species (Fig. 3 in Braithwaite, 1964). Implicit in the figure is a clear relationship between shape and ploidy in the sexual species, with the high‐ploidy spores having the higher length : width ratios. The apomict has the lowest ratio by far. Thus, new studies, addressing shape in other groups of ferns, including those in which some species are apomictic, may prove productive.

Finally, we hope that this work will inspire additional studies of the relationship between genome size and spore size in different clades. Our finding that spore volume is comparable across both trilete and monolete spores, but that the effect of genome size on spore size is greater for *Polystichum*, elicits curiosity about the nature of this relationship for other fern clades. It seems likely that genome size acts as a fundamental determinant of spore dimensions, but is shaped by other factors such as reproductive strategy, ecological preferences, and historical variation.

## Supporting information


**APPENDIX S1.** Spore measurements for all spores included in the analysis (in microns except for the ratio).

**APPENDIX S2.** Analysis of spore measurements and values for all accessions used in the present study (in microns except for the ratio).

## APPENDIX 1 Voucher information for *Adiantum* and *Polystichum* species used in this study. All vouchers are housed in the Pringle Herbarium (University of Vermont) unless indicated otherwise.

**Table:**

Species	Voucher specimen accession no.	Collection locality^b^	Geographic coordinates
*Adiantum aleuticum* (Rupr.) C. A. Paris	M. Southgate 302	Westfield, VT	44.8628659, −72.4169509
*A. aleuticum*	M. Southgate 418	Serpentine‐de‐Coleraine Ecological Reserve, QC	45.9927330, −71.3897020
*A. aleuticum*	M. Southgate 524	Serpentine‐de‐Coleraine Ecological Reserve, QC	45.9923429, −71.3839530
*A. pedatum* L.	M. Southgate 513	South Bolton, QC	45.1726170, −72.3763479
*A. pedatum*	M. Southgate 515	South Bolton, QC	45.1726170, −72.3763479
*A. pedatum*	M. Southgate 540	Goat Hill, West Nottingham, PA	39.7223019, −76.0804789
*A. viridimontanum* C. A. Paris	M. Southgate 169	Lowell, VT	NA
*A. viridimontanum*	M. Southgate 488	Melbourne, QC	45.6136279, −72.1024900
*A. viridimontanum*	M. Southgate 608	Troy, VT	NA
*Polystichum californicum* (D. C. Eaton) Diels	L. Ahart 20585	Butte Co., CA	39.808194, −121.724361
*P. californicum*	E. Alverson 2015‐66B	Lane Co., Umpqua National Forest, OR	43.640278, −122.651667
*P. californicum*	E. Alverson 2015‐66C	Lane Co., Umpqua National Forest, OR	43.640278, −122.651667
*P. californicum*	E. Alverson 2015‐66D	Lane Co., Umpqua National Forest, OR	43.640278, −122.651667
*P. imbricans* (D. C. Eaton) D. H. Wagner subsp. *curtum* (Ewan) D. H. Wagner	E. Alverson 2015‐15	San Diego Co., Cuyamaca Rancho State Park, CA	32.922319, −116.579339
*P. imbricans* subsp. *curtum*	E. Alverson 2015‐63B	Lane Co., Spencer Butte Summit, OR	43.983056, −123.095833
*P. dudleyi* Maxon	C. Rothfels 4613; UC	Marin Co., west end of Phoenix Lake, CA	37.95279, −122.58138
*P. dudleyi*	D. Barrington 1238	San Mateo Co., Portola State Park, CA	37.261018, −122.214593
*P. imbricans* subsp. *imbricans*	L. Ahart 20581	Butte Co., CA	39.551111, −121.208417
*P. imbricans* subsp. *imbricans*	C. Rothfels 4613; UC	Placer Co., near Middle Fork of American River, CA	39.0047167, −120.73305
*P. imbricans* subsp. *imbricans*	R. Barnard 3	Placer Co., near Middle Fork of American River, CA	39.006667, −120.7313833
*P. imbricans* subsp. *imbricans*	R. Barnard 5	El Dorado Co., El Dorado National Forest, CA	38. 669333, −120.5045667
*P. munitum* (Kaulf.) C. Presl	E. Alverson 2015‐64A	Spencer Butte Upper slope, OR	43.981111, −123.093333
*P. munitum*	E. Alverson 2015‐65A	Spencer Butte trailhead, OR	43.979722, −123.101944
*P. munitum*	E. Alverson 2015‐65B	Spencer Butte trailhead, OR	43.979722, −123.101944

Note

UC = University of California Herbarium, Berkeley, California, USA; NA

## APPENDIX 2 ANOVA of spore size and shape trait values as a function of species identity in *Polystichum*.

**Table:**

Spore trait	df	*F* value	*P* value
Average length	4, 225	87.55	<0.0001
Width	4, 225	62.57	<0.0001
Length : width	4, 225	1.632	0.167
Volume	4, 225	89.44	<0.0001

## APPENDIX 3 *P* values from Tukey post‐hoc tests of differences in spore size and shape trait values between all *Polystichum* taxon pair combinations (using *P. imbricans* subspecies names).

**Table:**

Species contrasts	Average length	Width	Length : width	Volume
*P. curtum‐P. californicum*	<0.0001	<0.0001	0.4624	<0.0001
*P. dudleyi‐P. californicum*	<0.0001	<0.0001	0.5621	<0.0001
*P. imbricans‐P. californicum*	<0.0001	<0.0001	0.2816	<0.0001
*P. munitum‐P. californicum*	<0.0001	<0.0001	0.9998	<0.0001
*P. dudleyi‐P. curtum*	<0.0001	<0.0001	0.9992	<0.0001
*P. imbricans‐P. curtum*	0.0011	0.0047	0.9734	0.0025
*P. munitum‐P. curtum*	<0.0001	<0.0001	0.5959	<0.0001
*P. imbricans‐P. dudleyi*	0.1571	0.3497	0.9991	0.3460
*P. munitum‐P. dudleyi*	0.3156	0.9716	0.6656	0.9909
*P. munitum‐P. imbricans*	<0.0001	0.0484	0.3797	0.0765

## APPENDIX 4 ANOVA of spore size and shape trait values as a function of species identity in *Adiantum*.

**Table:**

Spore trait	df	*F* value	*P* value
Average width	2, 89	157.5	<0.0001
Height	2, 89	113.8	<0.0001
Width : height	2, 89	7.038	0.00145
Volume	2, 89	87.25	<0.0001

## APPENDIX 5 *P* values from Tukey post‐hoc tests of differences in spore size and shape trait values between all *Adiantum* taxon pair combinations.

**Table:**

Species contrasts	Average width	Height	Width : height	Volume
*A. pedatum‐A. aleuticum*	<0.0001	0.000385	0.0381	0.0321
*A. aleuticum‐A. viridimontanum*	<0.0001	<0.0001	0.0577	<0.0001
*A. viridimontanum‐A. pedatum*	<0.0001	<0.0001	0.0423	<0.0001
